# Complex Interplay between Sphingolipid and Sterol Metabolism Revealed by Perturbations to the Leishmania Metabolome Caused by Miltefosine

**DOI:** 10.1128/AAC.02095-17

**Published:** 2018-04-26

**Authors:** Emily G. Armitage, Amjed Q. I. Alqaisi, Joanna Godzien, Imanol Peña, Alison J. Mbekeani, Vanesa Alonso-Herranz, Ángeles López-Gonzálvez, Julio Martín, Raquel Gabarro, Paul W. Denny, Michael P. Barrett, Coral Barbas

**Affiliations:** a Centre for Metabolomics and Bioanalysis (CEMBIO), Facultad de Farmacia, Universidad CEU San Pablo, Campus Montepríncipe, Boadilla del Monte, Madrid, Spain; b GSK I+D Diseases of the Developing World (DDW), Parque Tecnológico de Madrid, Tres Cantos, Madrid, Spain; c Wellcome Centre for Molecular Parasitology, Institute of Infection, Immunity and Inflammation, College of Medical, Veterinary and Life Sciences & Glasgow Polyomics, University of Glasgow, Glasgow, United Kingdom; d Department of Biosciences, Durham University, Lower Mountjoy, Durham, United Kingdom; e University of Baghdad, College of Science, Biology Department, Baghdad, Iraq

**Keywords:** Leishmania, lipid metabolism, mechanisms of action, metabolomics, miltefosine

## Abstract

With the World Health Organization reporting over 30,000 deaths and 200,000 to 400,000 new cases annually, visceral leishmaniasis is a serious disease affecting some of the world's poorest people. As drug resistance continues to rise, there is a huge unmet need to improve treatment. Miltefosine remains one of the main treatments for leishmaniasis, yet its mode of action (MoA) is still unknown. Understanding the MoA of this drug and parasite response to treatment could help pave the way for new and more successful treatments for leishmaniasis. A novel method has been devised to study the metabolome and lipidome of Leishmania donovani axenic amastigotes treated with miltefosine. Miltefosine caused a dramatic decrease in many membrane phospholipids (PLs), in addition to amino acid pools, while sphingolipids (SLs) and sterols increased. Leishmania major promastigotes devoid of SL biosynthesis through loss of the serine palmitoyl transferase gene (ΔLCB2) were 3-fold less sensitive to miltefosine than wild-type (WT) parasites. Changes in the metabolome and lipidome of miltefosine-treated L. major mirrored those of L. donovani. A lack of SLs in the ΔLCB2 mutant was matched by substantial alterations in sterol content. Together, these data indicate that SLs and ergosterol are important for miltefosine sensitivity and, perhaps, MoA.

## INTRODUCTION

Infectious diseases continue to cause great morbidity and mortality worldwide (1). New drugs are required and will need to be continuously replenished as resistance to antimicrobials increases. Understanding the mode of action (MoA) of currently available treatments against microbial diseases offers a means to highlight targets for new treatments. Metabolomics plays an important role in this discovery and development of new medicines for infectious diseases (1).

The leishmaniases are a spectrum of neglected tropical diseases caused by protozoa of the genus Leishmania. Individual species provoke different clinical manifestations, including visceral leishmaniasis caused by Leishmania donovani and Leishmania infantum (2) which is fatal if not treated. Existing therapeutic options are limited (3), so the search for alternative therapies continues. Two key developmental stages of Leishmania are used for *in vitro* studies of drug MoA: amastigotes and, more commonly, promastigotes. Promastigotes are the form found in the sand fly vector, while amastigotes exist in the mammalian host. The development of axenic cultures having physiological similarity to the macrophage resident forms in mammalian infections has made it possible to study amastigotes *in vitro* (2).

Metabolomics seeks comprehensive measurements of small molecules in a given system. However, the dynamic range in abundance and broad physicochemical diversity of metabolites is such that a single analytical platform is lacking. Here, a combined liquid chromatography-mass spectrometry (LC-MS) and capillary electrophoresis-mass spectrometry (CE-MS) approach was used to increase coverage of the Leishmania metabolome and applied to study the MoA of miltefosine, the first drug approved for oral treatment of leishmaniasis. Metabolomics has proven useful in drug MoA studies for Leishmania promastigotes (4–13) but so far not for L. donovani amastigotes. Leishmania mexicana amastigotes were recently studied using metabolomics to show that amastigote differentiation is associated with the induction of a distinct stringent metabolic state (14) in both lesion-derived and *in vitro* differentiated amastigotes.

Several suggestions have been made regarding the antileishmanial action of miltefosine, occurring either via induction of apoptosis-like death (15, 16) or disruption of metabolite transport (4, 17, 18). The uptake of miltefosine in Leishmania spp. is dependent on transmembrane lipid transporters, most notably the flippase LdMT and its accessory protein, LdRos, which are commonly lost with the selection of resistance (19). More recently, using cosmid-based functional cloning coupled with next-generation sequencing, genes involved in ergosterol biosynthesis and phospholipid (PL) translocation were suggested to contribute to resistance in L. infantum (20).

Metabolomic analyses of miltefosine-treated L. infantum strains showed a general depletion of intracellular metabolites (6), and similar studies in other Leishmania spp. demonstrated lipid remodelling (21–24). Biochemical modifications to different lipid classes have been reported in the membranes of miltefosine-treated L. donovani promastigotes (22). In addition to diminishing phosphatidylcholine (PC), miltefosine was found to double sterol composition as well (22). The effects of miltefosine treatment on lipid metabolism in promastigotes of L. infantum (6) have also been observed using metabolomics, although only lipid class rather than individual lipid species was resolved. Combining CE-MS to analyze polar and ionic metabolites and reversed-phase LC-MS to reveal specific lipidomic changes in L. donovani has allowed a more detailed analysis into the MoA in axenic amastigotes presented herein.

Substantial changes in sphingolipid (SL) and sterol metabolism were revealed in miltefosine-treated L. donovani promastigotes. SLs were first reported in L. donovani more than 20 years ago (25), and along with sterols, have since received attention in many species (26–28). Leishmania spp. obtain SLs via salvage or *de novo* synthesis (29), and they play important roles in differentiation, replication, trafficking, and virulence (29). The prominent, and most studied, SL identified is inositol phosphoceramide (IPC), although Leishmania spp. are believed to exhibit a complete and functional SL pathway involving both biosynthesis and degradation (28, 30). A more comprehensive analysis of different SLs may identify other key targets of SL metabolism for therapy. To study the viability of Leishmania spp. without SL synthesis, a mutant was created by deletion of the gene encoding the essential catalytic subunit of the serine palmitoyltransferase (ΔLCB2), the first and rate-limiting step in SL biosynthesis (28, 31). Surprisingly, this mutant was viable, indicating a dispensable role for SLs in Leishmania spp., unlike in the related parasite Trypanosoma brucei (32). It was suggested that the sterol composition of the Leishmania plasma membrane, where ergosterol replaces cholesterol as the primary membrane sterol, could enable this (32). Here, perturbations to Leishmania SL and sterol metabolism on miltefosine treatment are described, the interplay between these metabolite families is considered, and the role sterols play in drug sensitivity is proposed.

## RESULTS AND DISCUSSION

In order to define the optimal protocol for sampling/quenching/extracting/analyzing metabolites from L. donovani axenic amastigotes, a method was developed as described below and further in the supplemental material (File S1). The extraction procedure was optimized such that LC-MS and CE-MS analyses could be performed from single samples of as few as 1 × 10^7^ parasites (Fig. S1). For L. donovani, samples were treated with 4.47 μM (the observed 50% effective concentration [EC_50_] at 72 h, consistent with the literature [33]) or 13.41 μM (three times the observed EC_50_ at 72 h) miltefosine and harvested after 5 h or 24 h of exposure to observe the initial effects of the drug. For L. major, samples were treated with 10 μM or 30 μM miltefosine and harvested after 5 h of exposure. Dimethyl sulfoxide (DMSO) controls were prepared alongside treated samples at each time point for both species. The results from method development stages are detailed in the supplemental material: a comparison of methanol extraction and a more comprehensive extraction for lipidomics using LC-MS is shown in Fig. S2, profiles obtained using LC-MS and CE-MS analysis of different extractions are shown in Fig. S3, and the final optimized dual extraction procedure to obtain different extracts for LC-MS and CE-MS analysis from single samples is shown in Fig. S4.

### Metabolomic determination of Leishmania response to miltefosine.

With the exception of the aforementioned work on L. mexicana amastigotes (14), the few studies on drug MoA in Leishmania spp. have focused on the promastigote form (2–10). The aim of this research was to explore effects on metabolism of miltefosine in L. donovani axenic amastigotes and, as a result of these findings, in L. major promastigotes.

After verification of quality (Fig. S5), data were divided into separate sets, and differences between treated and untreated parasites were identified. Around 20 metabolites, including the drug itself, were detected only in the treated samples. These features (listed on the "Miltefosine related metabolites" tab in the supplemental tables file) were all found to elute with the drug and therefore were most likely enhanced by ionization of the drug. These could not be identified as endogenous lipids and were therefore assumed to be mass spectrometry derivatives of the drug itself. All were removed prior to multivariate analysis to avoid separation based on the presence or absence of drug alone. Identification was performed for metabolite features found to increase/decrease with treatment after 5 or 24 h of exposure, determined by a *P* value of <0.05 (Student's two-tailed *t* test, *n* = 6 per group) and a fold change of ±1.5 calculated for at least one of the comparisons made. Identification of metabolites found by CE-MS was confirmed by injection of authentic standards (as detailed in Table S1). Lipids detected using LC-MS were annotated considering chemical properties and elution order. Miltefosine treatment affected different metabolic classes, and the possible impact on MoA is discussed below, based on data presented in the supplemental tables, a description for which is given in the supplemental material.

### L. donovani axenic amastigotes.

**FIG 1:**
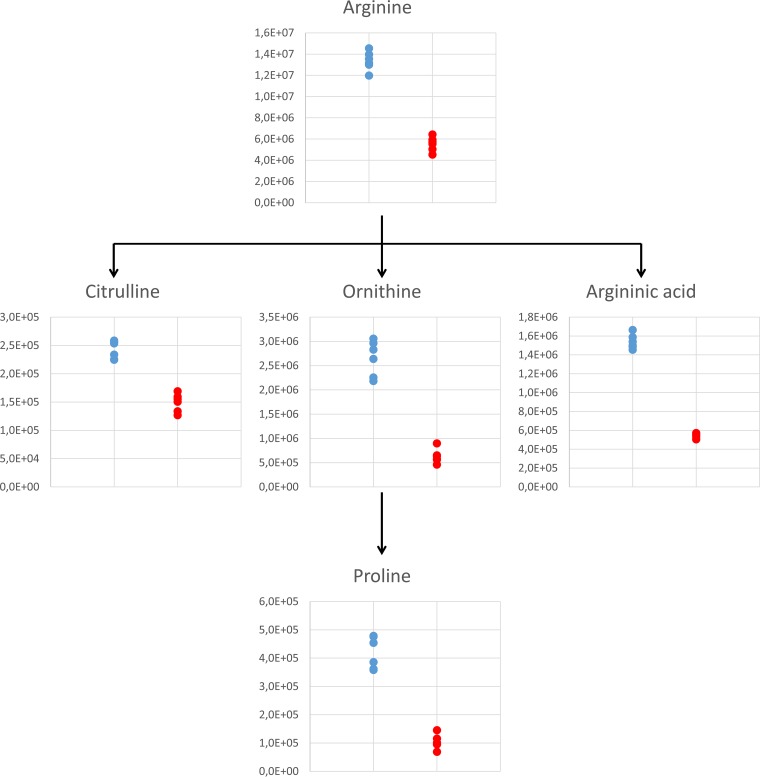
Effect of miltefosine on arginine metabolism observed in L. donovani axenic amastigotes after 24 h of exposure at the lower dose of 4.47 μM. Plots show peak area abundances detected in samples: untreated parasites are in blue, and parasites treated with 4.47 μM miltefosine are in red.

Miltefosine has been proposed to affect the transport of different metabolites (4, 17, 18). Consistent with previous literature (6), miltefosine induced decreases in the majority of internal metabolites detected by CE-MS, as shown in Table 1, which could be associated with impaired uptake. Figure 1 shows the abundances of metabolites associated with arginine metabolism detected in this study. Arginine is a precursor to polyamine biosynthesis and a protein building block. Its intracellular concentration is controlled by dedicated sensory protein transporters (10, 18), which have been suggested to be targets of miltefosine (4). Arginase, which catalyzes the hydrolysis of arginine to ornithine, also contributes to the intracellular concentration of arginine. The coordinated decrease in arginine and ornithine may indicate a reduction in precursor levels (through blocking arginine transport), a notion consistent with previously reported literature (34, 35). Another metabolite that shares the same mass but has a distinct migration time to citrulline was identified as argininic acid, which is considered an endpoint of arginine metabolism previously found in different Leishmania species, including L. donovani (36). Its concentration was substantially reduced with treatment at both time points and doses (up to 3-fold) in this research.

**TABLE 1:**
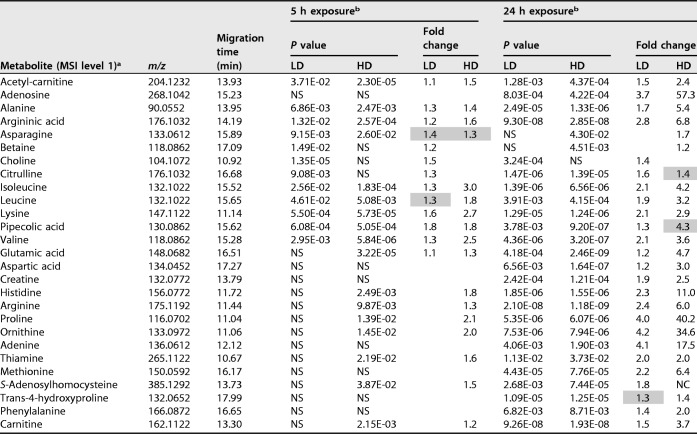
Metabolites identified in Leishmania donovani axenic amastigotes and significantly affected by miltefosine

a Metabolites identified in CE-MS analysis of Leishmania donovani axenic amastigotes as being significantly affected by miltefosine treatment in different doses/time points. All identifications have been determined at MSI (metabolomics standards initiative) level 1, as defined by the analysis of authentic standards.

b Calculated *P* values (Student's two-tailed *t* test [*n* = 6 per group]) and fold changes are shown for the lower dose (LD; 4.47 μM) and higher dose (HD; 13.41 μM) versus the untreated samples at the respective time point. Where *P* values were not significant (NS; *P* > 0.05), there were no fold changes to report. Fold changes are absolute; all are decreases except those highlighted in gray, which are calculated increases with miltefosine with respect to the untreated controls.

While data from CE-MS largely confirmed observations in the literature, the approach here has given a finer-grained view of the effect of miltefosine on lipids. Leishmania membrane lipids differ substantially in composition and function from those in mammals, making them important for viability and virulence as well as potential drug targets (29). Lipids have been analyzed previously in L. donovani (7, 22, 29, 37), as have changes in lipid metabolism connected to miltefosine treatment (21–23, 38, 39). Here, we demonstrate more detail on individual lipid species than has previously been reported.

In L. infantum, miltefosine was reported to alter 10% of the metabolome, purportedly due to compromised outer membrane integrity leading to lysis (6). Here, a general decrease in membrane PL abundance was observed in L. donovani axenic amastigotes (see Tables S2 to S4 for specific lipids). Miltefosine was reported by Zufferey et al. to inhibit PC biosynthesis, diminishing levels in L. donovani promastigotes leading to growth arrest (21). Phospholipase D activity was unaffected by the drug; hence, inhibition of the choline transporter was proposed to underlie the reduction in PC biosynthesis (21). A number of PCs and other PLs were found diminished in this study as well (Table S2).

**FIG 2:**
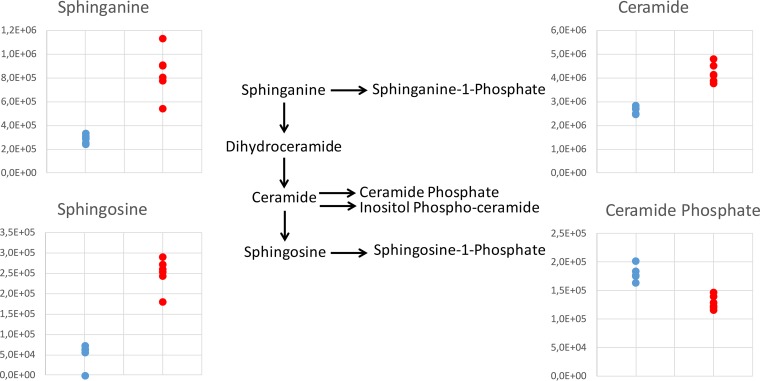
Effect of miltefosine on the SL biosynthetic pathway observed in L. donovani axenic amastigotes after 24 h of exposure at the lower dose of 4.47 μM. Plots show peak area abundances for key SLs detected in samples: untreated parasites are in blue, parasites treated with 4.47 μM miltefosine are in red. Sphinganine shown is the C_16_ form, ceramide shown is the 34:1 form, sphingosine shown is the 18:1 form, and ceramide phosphate shown is 26:1 form. Plots show trends representative of all detected SLs of their type. For full list of detected SLs, refer to Table S3.

Other considerable effects of treatment observed were increases in sterols and, to an even greater extent, SLs. To investigate this further, all filtered LC-MS data were scanned to identify peaks identifiable as SLs (even if the relative levels in treated and untreated parasites were not statistically different). Figure 2 shows the trends observed in SLs, where data are plotted for untreated parasites and parasites treated with the lowest dose of miltefosine at 24 h. A dramatic and significant (5-fold) increase in sphingosine abundance induced by miltefosine (*P* = 2 × 10^−6^) was mirrored by a 3-fold increase in sphinganine (*P* = 7 × 10^−4^). All detected ceramides were also substantially increased by 24 h, as shown and detailed in Table S3.

### L. major promastigotes.

Leishmania SL metabolism has been best studied in the promastigote form of L. major, where a ΔLCB2 mutant lacking the first enzyme of the biosynthetic pathway, serine palmitoyl transferase, is available (28, 31). The effects of miltefosine in these L. major promastigotes were therefore investigated. The efficacy of miltefosine was established against both the ΔLCB2 mutant and wild-type lines; the EC_50_ for the wild type was 6.83 μM, consistent with previous literature (33), while for the mutant, the EC_50_ was three times higher at 21.21 μM. The lipidome of these parasites revealed major differences between the wild-type and ΔLCB2 lines, and the effects of miltefosine were also compared. Lipids identified with marked differences in abundance between any experimental groups compared are detailed in Tables S5 to S7. As in L. donovani, miltefosine itself and around 20 other features were detected only in treated samples. Miltefosine was identified in both the wild type and ΔLCB2 mutant, with no significant difference in relative concentration (*P* = 0.09 for the lower dose and *P* = 0.45 at the higher dose). This demonstrated that the 3-fold resistance of the mutant was not due to inhibited import. As in L. donovani in this study and as reported in L. infantum previously (6), miltefosine caused substantial effects in levels of numerous PLs in L. major as well. This reduction may be due to reduced import of PLs or choline or to reduced *de novo* biosynthesis.

**FIG 3:**
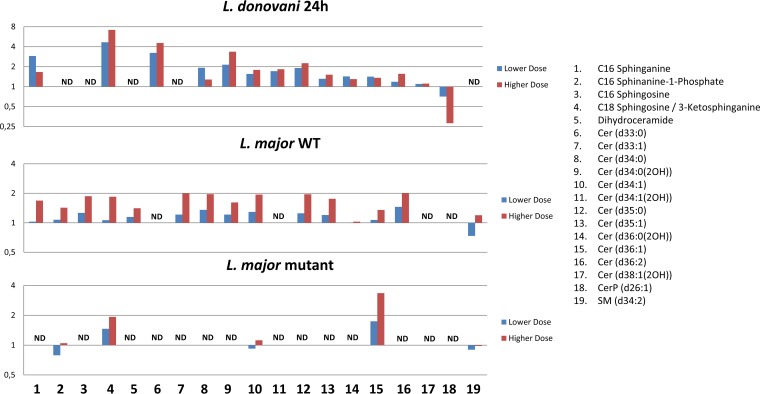
Fold changes in abundance of all SLs detected in L. donovani axenic amastigotes and/or L. major promastigotes, comparing treated parasites to untreated parasites. Lower-dose and higher-dose data are shown for each, corresponding to 4.47 μM and 13.41 μM, respectively, for L. donovani and 10 μM and 30 μM, respectively, for L. major. SLs that were not detected in a certain data set are marked with ND, while X denotes complete absence in drug-treated parasites and presence in untreated parasites.

The effects of miltefosine on L. major SLs were similar to those observed after 24 h of exposure in L. donovani amastigotes. Figure 3 shows the fold changes for both doses. As can be seen, some SLs were detected only in L. donovani or only in L. major. This may be due to species-specific differences and could even be due to differences in the mechanism of each species given that they cause different forms of leishmaniasis (L. donovani causing the visceral form and L. major the cutaneous form). The L. major ΔLCB2 mutants lack most SLs, in accordance with them being devoid of SL biosynthesis. Sphingosine and ceramide (d36:1), however, were detected, indicating that they are acquired from the media. Likewise, SM may also be derived from the media, since there is no evidence that Leishmania spp. synthesize SM, although they do possess SMase, which has been shown to be essential in degrading host-derived SM to promote parasite survival, proliferation, and virulence (40). The increase in SLs could indicate a stimulatory effect of miltefosine on biosynthesis or inhibition of a catabolic pathway, with the latter seeming more likely since the mutant also accumulates higher levels of the two SLs detected (SM and ceramide d36:1 upon treatment) (Fig. 3).

**FIG 4:**
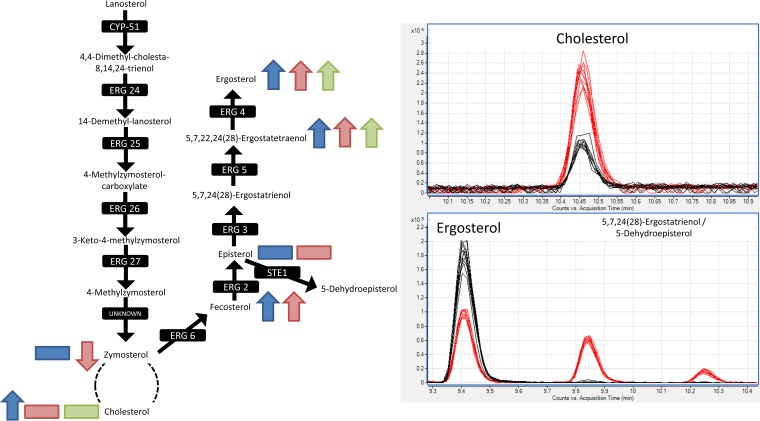
Ergosterol biosynthesis pathway. Sterols that increased, decreased, or were detected but with no change as a response to miltefosine exposure are shown on the pathway (L. donovani axenic amastigotes with 5-h drug exposure in blue, L. donovani axenic amastigotes with 24-h drug exposure in red, L. major wild-type promastigotes in green). In all cases, trends were seen for both low and high concentrations of miltefosine treatment. Chromatographic peaks shown for ergosterol, cholesterol, 5,7,24(28)-ergostatrienol, and 5-dehydroepisterol in L. major wild type (black trace) and ΔLCB2 mutants (red trace) to highlight the differences in sterol profiles between them. 5,7,24(28)-Ergostatrienol and 5-dehydroepisterol share the same logP, and therefore, it is not possible to distinguish to which peak (9.8 to 9.9 min or 10.2 to 10.3 min) these sterols correspond.

As in L. donovani, sterols were also increased by treatment in the L. major wild type, though not in ΔLCB2 mutants. The identification of sterols poses a particular challenge, since many in the pathway share identical masses. However, using the calculated LogP values, it was possible to identify each based on their elution order in the LC-MS data, as shown in File S2. Figure 4 shows the ergosterol biosynthesis pathway and highlights observed increases in L. donovani after 5 h (blue arrows) and 24 h (red arrows) of miltefosine exposure and in the L. major wild type after 5 h (green arrows) of miltefosine exposure. The trends were the same for both concentrations of the drug, except for fecosterol at the higher dose in L. donovani, which was increased, albeit not significantly.

Although sterol differences were not observed with treatment in ΔLCB2 mutants (Table S7), comparison to the untreated wild type revealed dramatic differences in sterol metabolism, particularly with respect to ergosterol and cholesterol. This alteration in sterol composition in the selection of the mutant is particularly noteworthy, since it enables the mutant to survive without SL synthesis, while the change in sterol composition may stabilize membranes in the face of miltefosine treatment, emphasizing the complex interplay between SL and sterol metabolism. As shown in the chromatographic peaks in Fig. 4, ergosterol levels were significantly reduced (approximately halved *P* = 5 × 10^−14^) in the ΔLCB2 mutant relative to the wild type, while cholesterol levels were around 3-fold more abundant (*P* = 8 × 10^−12^). Cholesterol is probably scavenged in Leishmania species (26). Ergosterol was significantly increased with treatment in the wild type and was of much lower abundance in the mutant, which exhibits a 3-fold reduced sensitivity to the drug. 5,7,22,24(28)-Ergostatetraenol, which precedes ergosterol in its synthetic pathway, is also dramatically more abundant in ΔLCB2 mutants than in the wild type, which suggests that the final step in ergosterol synthesis (catalyzed by erg4) is diminished in the mutant. Two further sterols, which share the same mass as ergosterol, 5,7,24(28)-ergostatrienol and 5-dehydroepisterol, were observed in the mutant but were below the limit of detection in the wild type. Their accumulation may also occur due to the reduced production in ergosterol biosynthesis later in the pathway.

The ΔLCB2 Leishmania mutants are viable, while the same enzyme is essential to African trypanosomes. This has been proposed to be due to Leishmania spp., depending on ergosterol as its primary sterol rather than cholesterol as in T. brucei (29). However, since the ΔLCB2 mutant exhibits much higher cholesterol and lower ergosterol concentrations than the wild type, the simplistic view of changes in cholesterol versus ergosterol appears to be inadequate to explain the essential nature of SL synthesis in T. brucei. Though the magnitude of this sterol balance is not as severe as in T. brucei, the retained viability in the absence of SL synthesis is likely to be due to other reasons. As observed in the L. major ΔLCB2 mutants, ergosterol reduction has been reported in a strain of L. infantum resistant to 200 μM miltefosine compared to in the wild type (41), although the mechanism of resistance was reported to be associated with mutations in the miltefosine transporter. In L. donovani promastigotes, membrane sterol depletion has been correlated with reduced sensitivity to miltefosine (42). In that study, the authors tested a hypothesis that lipid rafts could be involved in miltefosine action by destabilizing these microdomains through the depletion of sterols using either methyl-β-cyclodextrin (MCD) or cholesterol oxidase (CH-OX). Sterol depletion showed no significant effects on the viability of either the wild type or mutant; however, MCD treatment significantly decreased the susceptibility of the wild type to miltefosine (around 2-fold), although CH-OX depletion caused no significant effect. Since MCD has less specificity in sterol extraction than CH-OX, MCD is likely to deplete ergosterol and other sterols in addition to cholesterol, pointing to a possible link between ergosterol depletion and reduced miltefosine activity. Increases in ergosterol in L. donovani and L. major (wild type) coordinated with an increase in SLs reported here may also point toward a function of lipid microdomain complexes of sterols and SLs (27, 29, 42) in the effects of miltefosine on the parasites. Apoptosis has been proposed as an effect of miltefosine in tumor cells relating to lipid microdomains (43–45). Though the existence of apoptosis in Leishmania spp. has been challenged (46), various indications point to miltefosine inducing an apoptosis-like death in L. donovani promastigotes (16). It is clear that miltefosine treatment causes profound changes to the lipid contents of Leishmania amastigotes and promastigotes, and that alterations in lipid composition, such as a loss of SL biosynthesis and an accompanying change in sterol metabolism, impact this action of the drug.

### Conclusion.

A robust platform offering broad coverage of Leishmania metabolites using two complementary techniques was developed to study the miltefosine MoA. In addition to revealing the effects of miltefosine on internal metabolites and possible interference with membrane transport, many lipid species were shown to be perturbed by treatment, and importantly, SLs and sterols were found to increase. These findings, initially observed in L. donovani axenic amastigotes, were confirmed in L. major promastigotes for which a defined ΔLCB2 mutant, devoid of SL biosynthesis, was available. These mutant parasites lacked SLs, other than those derived from the culture medium, and sterol metabolism was drastically altered compared with the wild type. This lipidomic remodelling was associated with a 3-fold reduction in sensitivity to miltefosine. Coupled with the observation that sterol concentrations increase when both L. major and L. donovani wild-type parasites were exposed to miltefosine, a major role for these lipids in miltefosine resistance and, perhaps MoA, is indicated.

## MATERIALS AND METHODS

### Experimental design.

In order to define the optimal protocol for sampling/quenching/extracting/analyzing metabolites from L. donovani axenic amastigotes, a two-stage experiment was designed that (i) allowed a reproducible global profile of metabolites to be obtained and (ii) allowed the execution of the optimized protocol in the exploration of miltefosine MoA. It was necessary to optimize parasite seeding densities and harvesting numbers to work with 5-h and 24-h time points to obtain sufficient biomass for metabolomics analyses, while also ensuring log-phase growth (metabolic steady state) in parasites at the time of harvesting. Parasites were always seeded from log-phase cultures to minimize lag phase (particularly important at 5 h). Optimal seeding densities were determined to be 6.67 × 10^6^ parasites/ml for samples to be harvested at 5 h and 1.33 × 10^6^ parasites/ml for samples to be harvested at 24 h. Details of the method development are given in full in the supplemental material.

### Chemicals and reagents.

The axenic culture medium used in all experiments was prepared from one batch prepared “in-house” as described by Peña et al. (2). The culture medium used for the L. major wild type was Schneider's Drosophila medium (Sigma-Aldrich) supplemented with heat-inactivated fetal bovine serum (15%). All methanol used was high-performance liquid chromatography (HPLC) grade, and formic acid was analytical grade. These chemicals, in addition to formaldehyde solution and phosphate-buffered saline (PBS), were purchased from Sigma-Aldrich. Ultrapure water was obtained using a Milli-Q Plus 185 system (Millipore, Billerica, MA, USA).

### Sample collection and quenching of metabolism.

L. donovani strain 1S2D (WHO designation: MHOM/SD/62/1S-CL2D) (47) was cultured by cycling between promastigotes and axenic amastigotes using protocols from prior work (2). Briefly, the promastigote form was grown at 29°C, and amastigote forms were grown at 37°C with 5% CO_2_ in different media adapted by De Rycker et al. (48). For experiments with miltefosine, three T75 flasks were prepared (with 30 ml of culture at the appropriate densities for 5 h or 24 h, as described above) for each condition: nontreated parasites, parasites treated with the lower dose of miltefosine (4.47 μM), and parasites treated with the higher dose of miltefosine (13.41 μM).

At the time of sample harvesting, culture from each flask was divided equally into two 15-ml Falcon tubes, resulting in six replicate samples for each condition. Before the division, 50 μl of each culture was collected into Eppendorf tubes to which 50 μl formaldehyde was added, and samples were stored at 4°C to be counted later in order to record the exact number of parasites from each flask at the time of harvesting. At the time of harvesting and throughout the subsequent processes, samples were maintained at 4°C.

After the collection of culture, each sample was centrifuged at 1,500 × *g* at 4°C for 15 min, after which medium was decanted and parasites were washed in 2 ml PBS (maintained at 4°C); then, samples were transferred to 2-ml Eppendorf tubes. From each sample, 10 μl was taken, fixed with 10 μl of formaldehyde, and stored at 4°C to be counted later in order to record the exact number of parasites in each sample immediately before quenching. Samples were subsequently centrifuged at 1,500 × *g* at 4°C for 15 min, PBS was decanted, and 200 μl ice-cold methanol was added to each sample; samples were immediately stored at −80°C until extraction and metabolomics analysis. Figure S1 shows the workflow for the developed method for sampling.

Leishmania major parasites (MHOM/IL/81/Friedlin; FV1 strain) and a mutant in which the catalytic subunit of serine palmitoyltransferase had been deleted by homologous recombination (ΔLCB2) (31) were cultured as log-phase promastigotes at 26°C. Samples were prepared for metabolomics as described for L. donovani with the appropriate miltefosine doses described above, except 10 μM and 30 μM were used for the lower and higher doses of miltefosine, respectively.

### Metabolite extraction.

Extraction blanks were prepared following all stages of extraction. On the day of analyses, metabolites were extracted and supernatants analyzed by LC-MS (and for all L. donovani samples in CE-MS as well). Samples were prepared by first evaporating extracts to dryness using a speed vacuum concentrator (Eppendorf, Hamburg, Germany), after which 200 mg of 425- to 600-μm acid-washed glass beads was added. Then to L. donovani samples, 575 μl of 100% methanol was added, before which samples were vortexed for 10 min and placed in a tissue lyzer for 30 min at 50 Hz. Finally, samples were centrifuged at 16,000 × *g* at 4°C for 10 min, and 80 μl of the resulting supernatants was collected into LC-MS vials to be analyzed directly. To the remaining samples (for CE-MS), 165 μl of water was added; they were vortexed for 30 min, centrifuged at 16,000 × *g* at 4°C for 10 min, evaporated to dryness, and resuspended in 100 μl of water containing 0.2 M methionine sulfone (internal standard), after which 0.1 mM formic acid was added to each. Quality control (QC) samples for LC-MS and CE-MS were prepared by collecting 10 μl from each sample into a single pool. For L. major, only LC-MS extracts were prepared; therefore, after evaporation, 120 mg of 425- to 600-μm acid-washed glass beads and 350 μl methanol were added for extraction, and 270 μl of the resulting supernatants following extraction was collected into LC-MS vials to be analyzed directly, from which 30 μl was subtracted from each into a pool.

### Analysis of extracts by LC-MS and CE-MS.

For each analysis, extraction blanks were injected at the start of the analysis, followed by eight injections of the QC sample in order to ensure system stability; samples were then analyzed in random order, with the QC sample injected after every sixth sample until the end of the analysis. All instrumentation was from Agilent Technologies. For LC-MS, a 1290 infinity LC equipped with reverse-phase column (Zorbax Extend C_18_, 50 by 2.1 mm, 3 μm; Agilent) was coupled to a 6550 Q-TOF MS with electrospray ionization source and operated in both positive and negative mode. For CE-MS, the instrument consisted of a 7100 CE coupled to a 6224 TOF MS operated in positive mode. Details of the analytical procedures based on previously published methods (49, 50) are given in the supplemental material.

### Data analysis and feature identification.

Data from both platforms were processed using recursive analysis in MassHunter Profinder (B.06.00; Agilent) software, as detailed in the supplemental material. Data were reprocessed considering ions such as [M+H]^+^ and [M+Na]^+^, with neutral water loss and a maximum permitted charge state that was double. Alignment was performed based on *m*/*z* and retention time (RT) similarities within the samples. The parameters applied were 1% for the RT window and 20 ppm for mass tolerance.

Data treatment consisted of filtering based on quality, following the same procedure for each data set (L. donovani, LC-MS positive-ion mode, LC-MS negative-ion mode, CE-MS positive-ion mode; L. major, LC-MS positive-ion mode, and LC-MS negative-ion mode). Data were filtered based on quality using a quality assurance procedure described previously (QA+) (51). This involved retaining features present in QC samples at a rate of 80% or absent in QC samples (defined as presence <20%). For features present in QC samples, only those with relative standard deviations (RSDs) of <30% were kept, and for those that were absent, the RSD was not calculated. Then, for each comparison separately (5 h, 24 h, wild type, or ΔLCB2 mutant), features were further filtered to keep only those present in at least five out of six of the replicates from one of the groups simultaneously compared (resulting in a slightly different but relevant data set for each comparison).

First, L. donovani multivariate analysis was employed to observe the stability in each analysis (LC-MS in positive- and negative-ionization modes or CE-MS) as a whole and then for each time point separately to investigate the effect of the drug on the parasite metabolome. To probe specific questions on the effect of miltefosine at different time points or doses, fold changes and *P* values were calculated in order to assess the degree of significance of any difference observed in the raw data.

All significantly different metabolite features between untreated and treated parasites at any dose, as determined by a *P* value of <0.05 (Student's two-tailed *t* test, *n* = 6 per group) and a fold change of ±1.5, were identified. Identification was performed by searching *m/z* against Metlin (http://metlin.scripps.edu) and Lipid Maps (http://lipidMAPS.org), considering the same adducts as those described for data reprocessing. Annotations were assigned to *m/z* values for metabolite features taking into consideration (i) mass accuracy (maximum mass error, 10 ppm), (ii) isotopic pattern distribution, (iii) the possibility of cation and anion formation, and (iv) adduct formation. This method of enhanced annotation was based on our previously published work (49). Where possible, identifications were compared by retention time order to standards analyzed in-house. For CE-MS, definitive identifications were made for a number of metabolites through an analysis of authentic standards analyzed under the same conditions as the experiment, whereby samples were analyzed again followed by the same samples spiked with authentic standards to prove the identity.

All data analyzed during this study are included in this article and in the tables in the supplemental material.

## Supplementary Material

Supplemental material
