# Supplementary material for: Complex Interplay between Sphingolipid and Sterol Metabolism Revealed by Perturbations to the Leishmania Metabolome Caused by Miltefosine
Source: Antimicrob Agents Chemother. 2018 Apr 26;62(5):e02095-17. doi: 10.1128/AAC.02095-17 (PMC5923112; doi:10.1128/AAC.02095-17)
Supplement: Supplemental material [file supp_62_5_e02095-17__index.html]

Supplemental material 

# Complex Interplay between Sphingolipid and Sterol Metabolism Revealed by Perturbations to the Leishmania Metabolome Caused by Miltefosine

## Supplemental material

- Supplemental file 1 -

  Supplemental text and figures

  PDF, 2.0M
- Supplemental file 2 -

  Supplemental tables

  XLSX, 229K
