# Supplementary material for: Complex Interplay between Sphingolipid and Sterol Metabolism Revealed by Perturbations to the Leishmania Metabolome Caused by Miltefosine
Source: Antimicrob Agents Chemother. 2018 Apr 26;62(5):e02095-17. doi: 10.1128/AAC.02095-17 (PMC5923112; doi:10.1128/AAC.02095-17)

A complex interplay between sphingolipid and sterol metabolism revealed by perturbations to the *Leishmania* metabolome caused by miltefosine

Emily G. Armitage[1,2,3], Amjed Q. I. Alqaisi[4,5], Joanna Godzien[1], Imanol Peña[2], Alison J. Mbekeani[4], Vanesa Alsonso-Herranz[1], Ángeles López-González[1], Julio Martín[2], Raquel Gabarro[2], Paul W. Denny[4]*, Michael P. Barrett[3]* and Coral Barbas[1]*

1. Centre for Metabolomics and Bioanalysis (CEMBIO), Facultad de Farmacia, Universidad CEU San Pablo, Campus Montepríncipe, Boadilla del Monte, 28668 Madrid, Spain

2. Tres Cantos Open-Lab Foundation, GlaxoSmithKline, Parque Tecnológico de Madrid, Calle de Severo Ochoa 2, 28760 Tres Cantos, Madrid, Spain

3. Wellcome Centre for Molecular Parasitology, Institute of Infection, Immunity and Inflammation, College of Medical, Veterinary and Life Sciences & Glasgow Polyomics, University of Glasgow, Glasgow, UK

4. Department of Biosciences, Durham University, Lower Mountjoy, Stockton Road, Durham, DH1 3LE, UK

5. University of Baghdad, College of Science, Biology Department, Baghdad, Iraq

*** Corresponding authors**

SUPPLEMENTARY INFORMATION 1

Optimisation of Leishmania sampling for metabolomics analysis

In order to define the optimal protocol for sampling, quenching, extracting and analysing metabolites from *Leishmania donovani* axenic amastigotes, a method was developed as described in the method section of the main article, supplemented by details below. Figure 1S shows the workflow for the developed method of sampling *Leishmania* parasites. Six biological replicates were prepared from seeding at a density of 1.33 × 10^6 parasites/mL and harvesting at 24 h. This experiment was designed to compare two extraction methods: utilising methanol or methanol:water 3:1 (*v/v*) analysed in both LC-MS and CE-MS. The results from this led to the optimisation of a double extraction method on a single sample to obtain samples for both analytical platforms. This was applied to study miltefosine mechanism of action in both *L. donovani* axenic amastigote samples and in *L .major* promastigote samples.

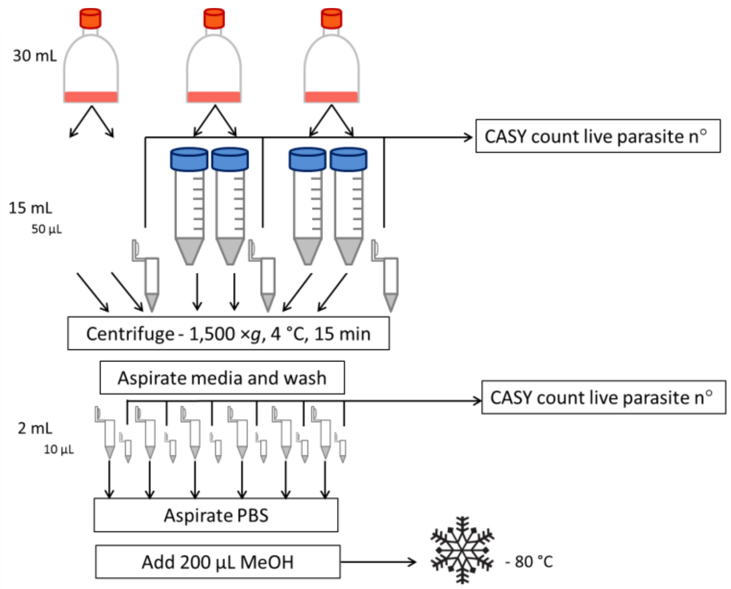

Figure 1S: Optimal procedure for sample collection, adaptable for different harvest times and corresponding initial starting densities of parasites, leading to six individual biological samples.

Method development analytical procedures

Metabolite extraction

For each type of extraction, extraction blanks were prepared using Eppendorf tubes from the same batch as those used for samples and following all stages of extraction. On the day of analysis, metabolites were extracted and resulting extracts were analysed by LC-MS (and for all *L. donovani* samples CE-MS). For the method development experiment, each sample was vortex mixed and split into two equal aliquots to which the different proportions of methanol were to be added. Samples were evaporated to dryness using a speed vacuum concentrator (Eppendorf, Hamburg, Germany), after which 200 mg of 425-600 µm acid-washed glass beads were added. Then, to the method development samples, 575 µL of either methanol or methanol:water (3:1) prepared with ultrapure water (maintained at 4 °C) was added. Samples were vortex mixed for 10 min and placed in a tissue lyzer for 30 min at 50 Hz. Finally samples were centrifuged at 16,000 × *g* at 4 °C for 10 min and supernatants were collected into vials for LC-MS and into Eppendorf tubes for CE-MS. These were then evaporated to dryness using the speed vacuum concentrator and once dry, 100 µL of water containing 0.2 mM methionine sulfone used as an internal standard and 0.1 M formic acid was added to each.

Analysis of extracts by LC-MS and CE-MS

Method development samples were analysed in a sequence starting with three injections of the 100 % methanol extraction blank, followed by eight injections of the first methanol extract sample to equilibrate and stabilise the system, then each of the six replicate samples in turn. After, the same sequence for methanol water samples was followed (extraction blanks, injections of an extract to obtain equilibrium and stability in the system and injections of each of the six biological replicates). All instrument settings for LC-MS and CE-MS were the same as described in the main article.

Data treatment

Following data processing as described in the main article, data were filtered separately for each extraction method including the respective extraction blank. The first stage of filtering was performed to keep only features present in at least five of the six replicates, secondly features were filtered to keep only those with relative standard deviations (RSD) < 30 % and ensuring that all features were absent or present in negligible abundance in the extraction blanks.

Results from method development

From preliminary experiments it was observed that the richest information could be obtained through LC-MS and CE-MS by extraction solvents methanol and methanol:water (3:1 *v/v*). A comparison for LC-MS was also made between methanol and a dual extraction of methanol:MTBE (2:1 *v/v*) followed by methanol:water (2:1 *v/v*) in order to explore the potential of the latter in improving the extraction of lipids and if overall it led to obtaining a greater number of reliable biological features in the profile. Three replicates of each were compared in addition to extraction blanks for each method and data were processed as described for all other samples. Data were filtered separately for each method of extraction to keep only those features that were present across all three replicates. Data were further filtered in each case to include only those features whose average abundance was greater in samples than blanks. The final metabolite feature lists were compared to observe which method gave a wider coverage of reproducible features. Figure 2S shows a Venn diagram of reproducible features obtained through each method whereby 198 were common, 436 were unique to the methanol extraction and 221 were unique to the dual method. Biologically feasible annotations could be given to the majority of peaks from each of the extractions, however, a greater whelm of biological information could be obtained with the methanol extraction and therefore this was chosen.

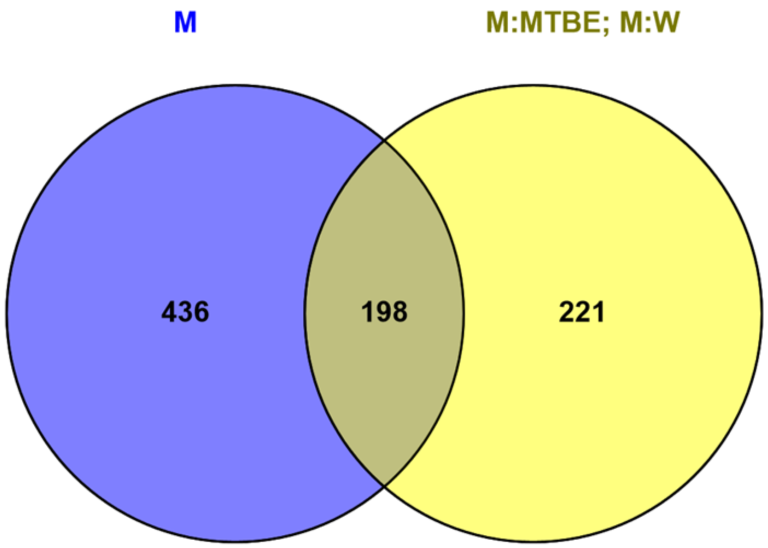

Figure 2S: Comparison of reproducible metabolite features obtained from the LC-MS analysis of samples extracted with methanol (M) or with a dual method involving methanol:MTBE (2:1 *v/v*) then methanol:water (2:1 *v/v*) - M:MTBE; M:W.

Methanol and methanol:water (3:1) extractions were subsequently tested in both CE-MS and LC-MS in order to devise a final method for the analysis of *Leishmania* axenic amastigotes. Figure 3S shows the best profiles obtained from CE-MS and LC-MS (in positive and negative ionisation modes) that were methanol for LC-MS and methanol:water (3:1) for CE-MS. Also in this figure, Venn diagrams highlighting the number of reproducible metabolic features obtained from each extraction measured in each platform (determined as those present in five out of six replicates, with RSD < 30 % and with signal higher than that or the respective blank).

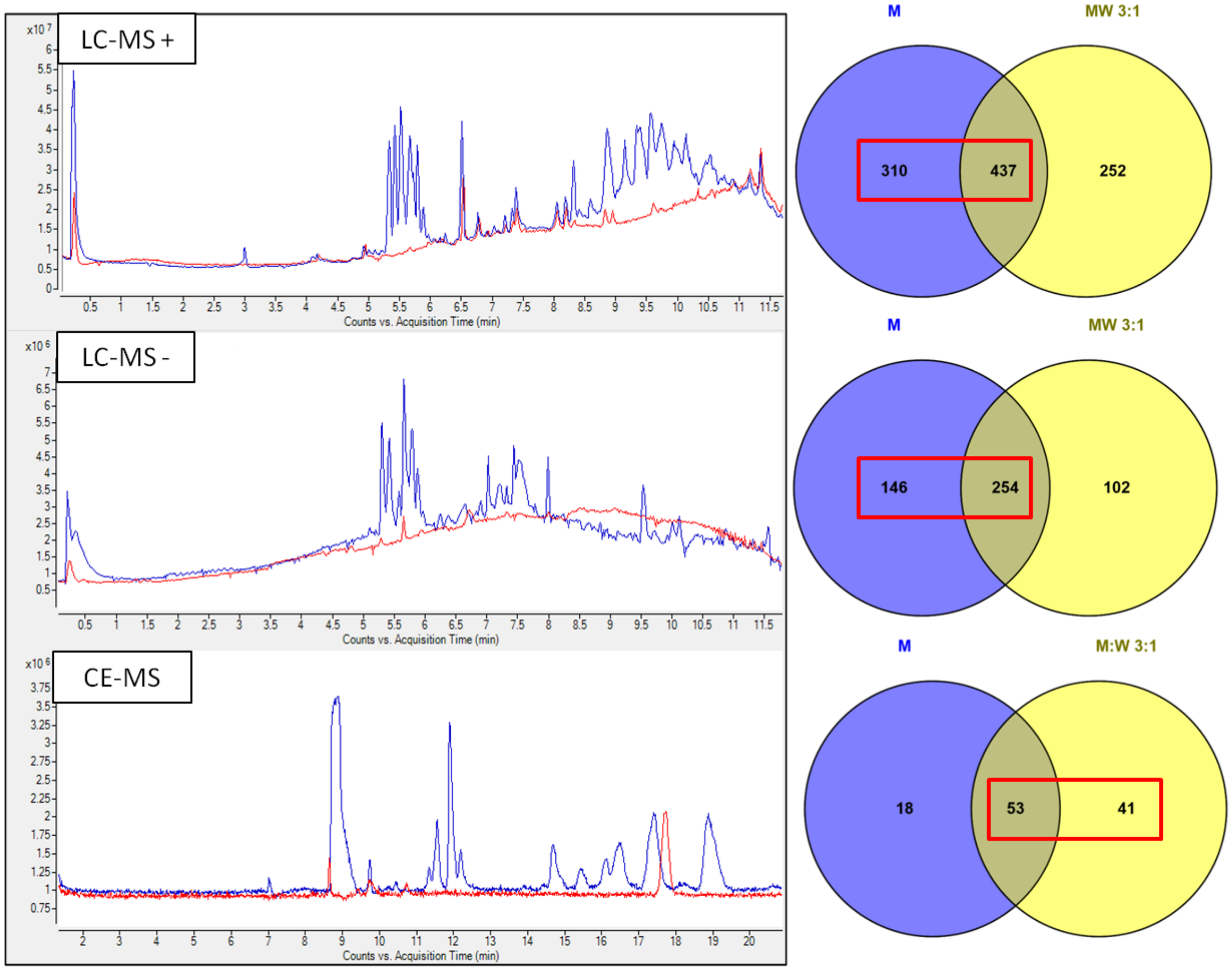

Figure 3S: Total ion chromatogram (LC-MS) or electropherogram (CE-MS) profiles for axenic amastigotes extracted with methanol - M (LC-MS) or methanol:water (3:1) - M:W 3:1 (CE-MS) and venn diagrams highlighting the numbers of metabolic features present in 5 out of 6 replicate samples with relative standard deviations (RSD) < 30%

and exhibiting signals greater than in the respective extraction blanks. In the profiles, one replicate sample is shown (blue) in addition to the respective extraction blank (red).

It was clear from this that different extractions are necessary to obtain optimal information from both platforms, and therefore, rather than choosing one as a compromise between the two platforms as a way to analyse one single sample in both, the method was further developed to include both extractions from a single sample of parasites. This reproducible method for sample collection, preparation (metabolic quenching and extraction) and analysis was devised following the workflow as shown in Figure 4S. This was the method applied in the second experiment - the proof of principle experiment - where the parameters selected for each step were those detailed in the materials and method section.

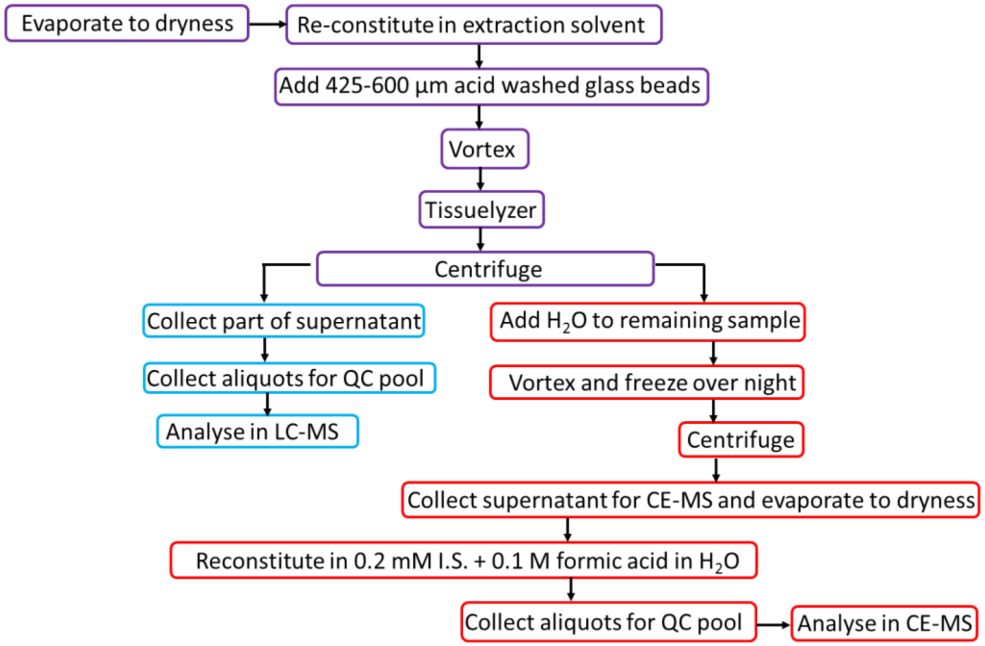

Figure 4S: Pipeline overview for the optimised dual extraction method from a single sample of parasites previously quenched in methanol and stored at -80 °C until analysis.

Control of parasite numbers in *L. donovani* experiments

As part of the sample collection procedure (Figure 1S), parasite number was recorded before harvesting and following washing in order to control the final number of parasites per sample. After storage of these samples for metabolomics analysis, samples collected for counting were analysed using the CASY® cell counter and the total number of parasites in each sample was recorded. Negligible differences were recorded between samples. At 5 h, the relative standard deviation (RSD) between final counts was 2.3 % considering samples of all three conditions (non-treated, treated with 4.47 µM or treated with 13.41 µM miltefosine) and 1.96 % considering only non-treated and 4.47 µM miltefosine. At 24h, the RSD between final counts was 23 % considering samples of all three conditions but only 0.58 % when those considering non-treated and treated at 4.47 µM miltefosine. This increased variability in parasite number when considering the higher dose and higher time-point was due to increased death which was not observed for all other time-point/dose combinations. Therefore the earlier 5h time-point with both doses and the lower dose at 24h are those are used to elucidate miltefosine MoA before the onset of parasite death.

Analysis of extracts by LC-MS (miltefosine MoA metabolomics)

Extraction blanks were injected at the start of the analysis followed by eight injections of the QC sample in order to ensure system stability, before the injection of samples analysed in a random order with the QC injected after every sixth sample until the end of the analysis.

The instrument consisted of a liquid chromatography system complete with degasser, binary pump and autosampler (1290 Infinity, Agilent). Different volumes of sample (1 µL for positive mode analyses and 2 µL for negative) were applied to a reverse-phase column (Zorbax Extend C_{18} 50 × 2.1 mm, 3 µm; Agilent), which was maintained at 60 °C during the analysis. The system was operated in positive and negative ion mode at a flow rate of 0.6 mL/min with solvent A (water with 0.1% formic acid) and solvent B (acetonitrile with 0.1% formic acid). The gradient was: 5 % B (0.0-1.0 min), 5 to 80 % B (1.0-7.0 min), 80 to 100 % B (7.0-11.5 min) and 100 to 5 % B (11.5-12.0 min), keeping the re-equilibration at 5 % B for 3 min (15.0 min of total analysis time). Data were collected in positive and negative ESI mode in separate analyses on a QTOF (Agilent 6550 iFunnel). Both ion modes were operated in full scan mode (m/z 50 to 1,000 in positive and m/z 50 to 1,100 in negative ion mode). In each the capillary voltage was 3,000 V, the scan rate was 1.2 spectra/s, the gas

temperature was 250°C, the drying gas flow 12 L/min and the nebulizer 52 psi. The MS-TOF parameters for positive ion mode were: fragmentor 225 V, skimmer 65 V and octopole radio frequency voltage (OCT RF Vpp) 750 V. MS-TOF parameters were the same in negative ion mode, except the scan rate which was 1.0 spectra/s and the capillary voltage which was 4,000 V. During the positive analysis, two reference masses were used: *m/z* 121.0509 ($[C_5H_4N_4+H]^+$) and *m/z* 922.0098 ($[C_{18}H_{18}O_6N_3P_3F_{24}+H]^+$) as well as in negative: *m/z* 112.9855 ($[C_2O_2F_3-H]^-$) and *m/z* 1033.9881 ($[C_{18}H_{18}O_6N_3P_3F_{24}+TFA-H]^-$). They were continuously infused into the system to allow constant mass correction. Samples were injected at 1 µL and 2 µL volumes for positive and negative ion modes respectively.

Analysis of extracts by CE-MS (miltefosine MoA metabolomics)

The sequence of analysis for CE-MS was the same as that described above for LC-MS.

The instrument consisted of a capillary electrophoresis (7100 Agilent) coupled to a TOF Mass Spectrometer (6224 Agilent) equipped with an ESI source, controlled by Mass Hunter Workstation Data Analysis (B.06.01, Agilent). The separation occurred in a fused-silica capillary (Agilent) (total length, 100 cm; internal diameter, 50 µm). All separations were carried out in normal polarity with a background electrolyte containing 1 M of formic acid solution in 10 % methanol (*v/v*) at 20 °C. In our laboratory, new capillaries are pre-conditioned with a flush of 1.0 M NaOH for 30 min followed by MilliQ® water for 30 min and background electrolyte for 30 min (although only one capillary was used in the analysis of all samples for this research). Before each analysis, the capillary was conditioned with a flush of background electrolyte for 5 min. The sheath liquid (6 µL/min) was MeOH:H_2O (1:1) containing 1.0 mM formic acid with two references masses: *m/z* 121.0509 ($[C_5H_4N_4+H]^+$) and *m/z* 922.0098 ($[C_{18}H_{18}O_6N_3P_3F_{24}+H]^+$), which allowed correction and higher mass accuracy in the MS. Samples were hydrodynamically injected at 50 mBar for 50 s. The stacking was carried out by applying background electrolyte at 100 mBar for 10 s. The separation voltage was 30 kV, the internal pressure was 25 mBar and the analyses were carried out in 35 min. The MS parameters were: fragmentor 100 V, skimmer 65 V, octopole 750 V, drying gas temperature 200 °C, flow rate 10 L/min and capillary voltage 3,500 V. Data were acquired in positive mode with a full scan from *m/z* 85 to 1,000 at a rate 1.41 scan/s.

Data analysis and feature identification (miltefosine MoA metabolomics)

Data from both CE-MS and LC-MS experiments were processed in the same way, using recursive analysis in Mass Hunter Profinder (B.06.00, Agilent) software. Recursive analysis was performed using two sequential processing stages: naïve reprocessing through Molecular Feature Extraction (MFE) and target reprocessing through Find by Ion (FbI). MFE was used to find co-eluting ions that are linked (related to charge-state envelope, isotopic distribution, and/or the presence of adducts and dimers, as well as potential neutral loss of molecules) and to sum all ion signals into one value defined as a feature. Subsequently, compounds were aligned and reviewed before performing FbI, which was employed to find from the list of features generated through MFE, any signals that were missed in some samples due to isotope and/or adduct abundance. This enabled a huge reduction on the number of missing values arising from data reprocessing errors, especially for low abundant features. Data were reprocessed considering ions such as $[M+H]^+$ and $[M+Na]^+$, neutral water loss and the maximum permitted charge state was double. Alignment was performed based on m/z and RT similarities within the samples. Parameters applied were 1 % for the RT window and 20 ppm for mass tolerance.

Assessment of data quality – *L. donovani* (miltefosine MoA metabolomics)

Data quality was first assessed by visualising the spread of biological samples and QC samples in PCA. Scores plots for each analysis (CE-MS and LC-MS performed in both positive and negative ion modes) are presented in Figure 5S. In each plot, QC samples being a pool of each sample are centrally placed with low intra-variability compared to biological samples, indicative of successful analyses.

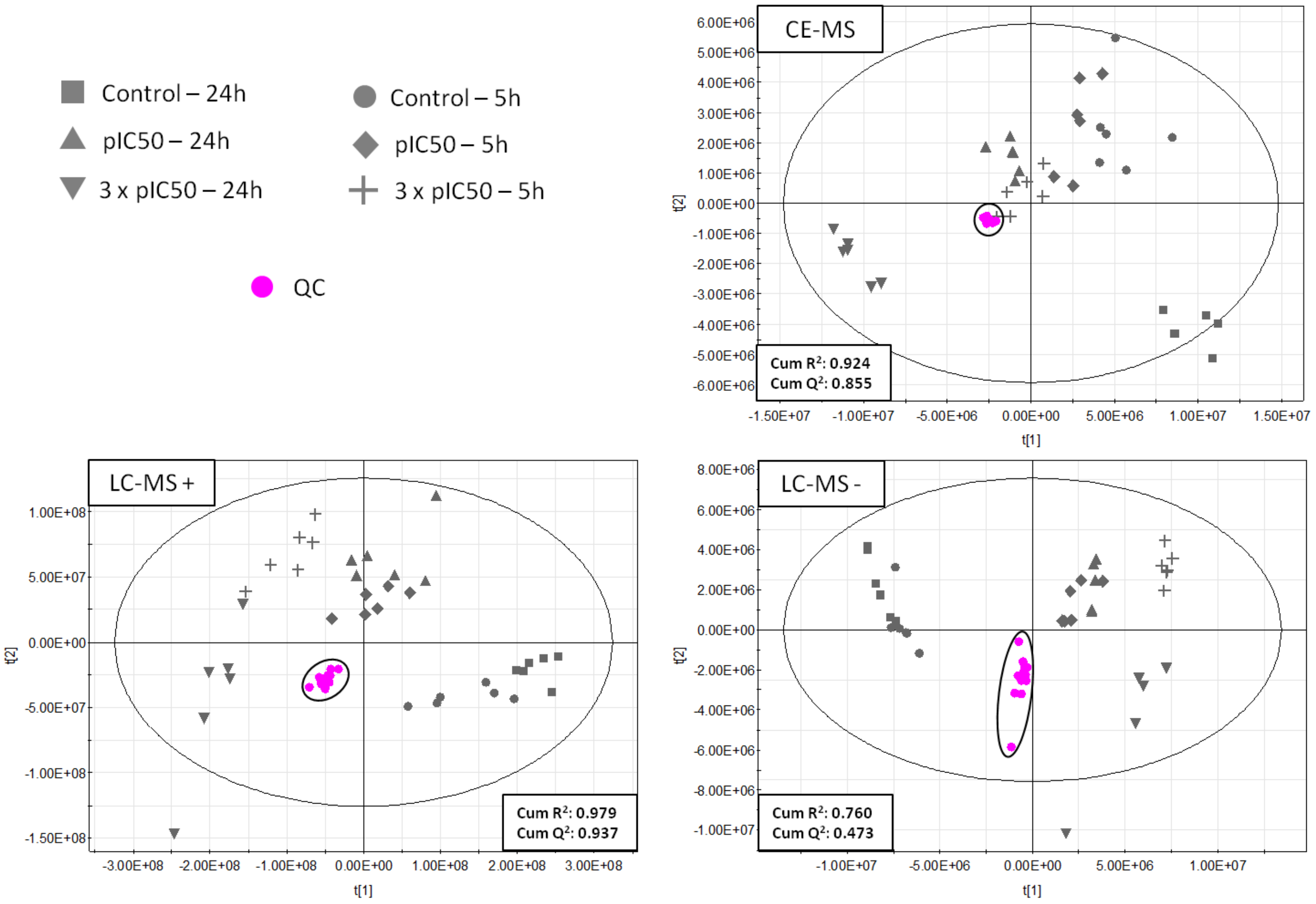

Figure 5S: PCA scores plots for each analysis - CE-MS, LC-MS (positive ion mode) and LC-MS (negative ion mode). All models were performed on mean centred data and the cumulative R^2 and Q^2 are shown for each model.

Supplementary Tables

Data are made available in supplementary tables:

Table 1 – *L. donovani* (CE-MS)

Table 2 – *L. donovani* Phospholipid

Table 3 – *L. donovani* Sphingolipid

Table 4 – *L. donovani* Sterols

Table 5 – *L. major* Phospholipid

Table 6 – *L. major* Sphingolipid

Table 7 – *L. major* Sterols

Additional table: Miltefosine and related metabolites

Each table details the metabolite/lipid identification/annotation, the m/z detected experimentally, the retention (LC) or migration (CE) time detected experimentally, the adduct of metabolite/lipid detected experimentally, the abundance detected in each individual sample (6 replicates for each group), the p-value calculated between treated and un-treated samples and the associated fold change calculated. The *p*-values were calculated in excel based on two-tailed t-test assuming unequal variance. Fold changes were calculated by dividing average abundance of treated samples by average abundance of un-treated samples as detected. When two or less samples per group contained missing values, these were not considered zero in *p*-value and fold change calculations. When a feature was completely absent from a group, p-value and fold change were not calculated (denoted NC).

SUPPLEMENTARY INFORMATION 2

Leishmania Sterols

Evidence to support annotations

Experimental *m/z* 381.3541 corresponding to water loss of 398.3549, mass of three sterols in pathway (highlighted)

4-methylzymosterol was discounted since it has no possibility for neutral loss of water in structure. Two peaks in each species determined to be first Fecosterol then Episterol determined from on LogP calculations.

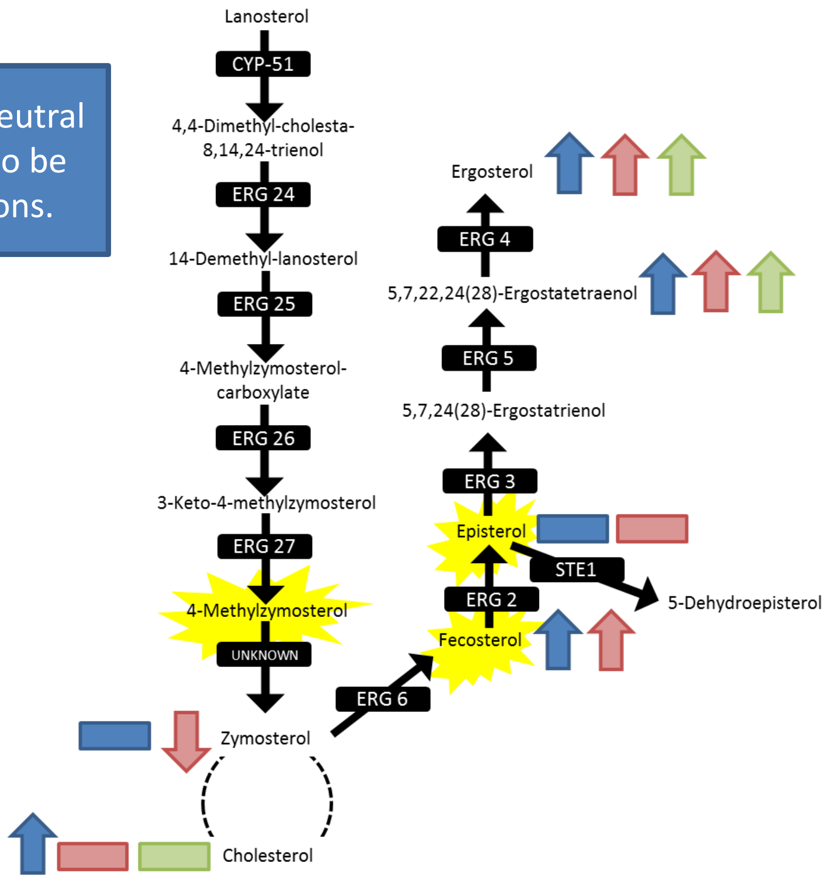

4-methylzymosterol **Fecosterol** **Episterol**

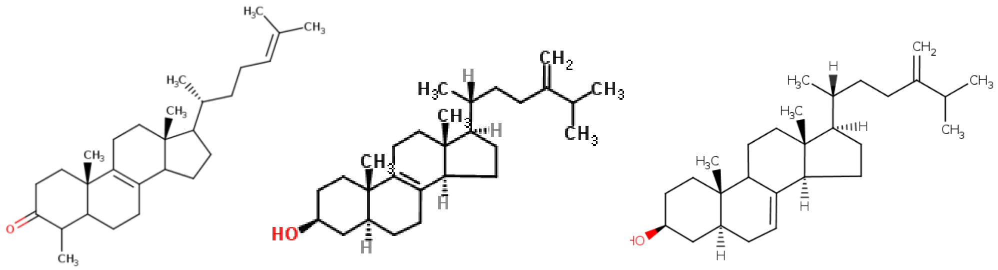

Fecosterol		
logP	6.65	ALOGPS
logP	7.01	ChemAxon

Episterol		
logP	7.16	ALOGPS
logP	7.05	ChemAxon

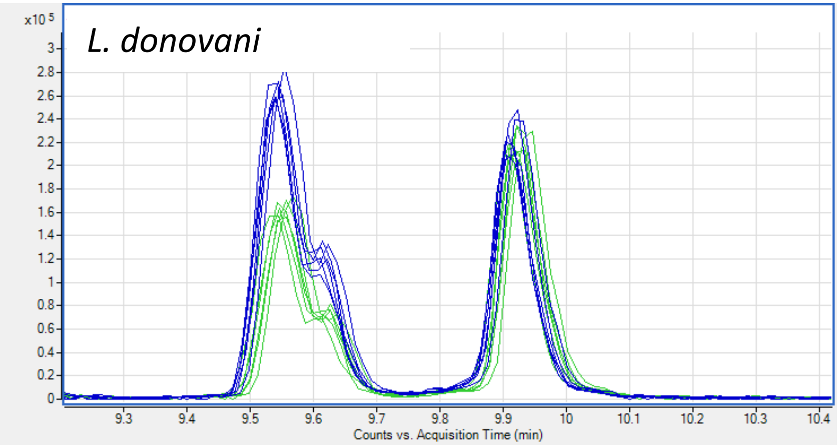

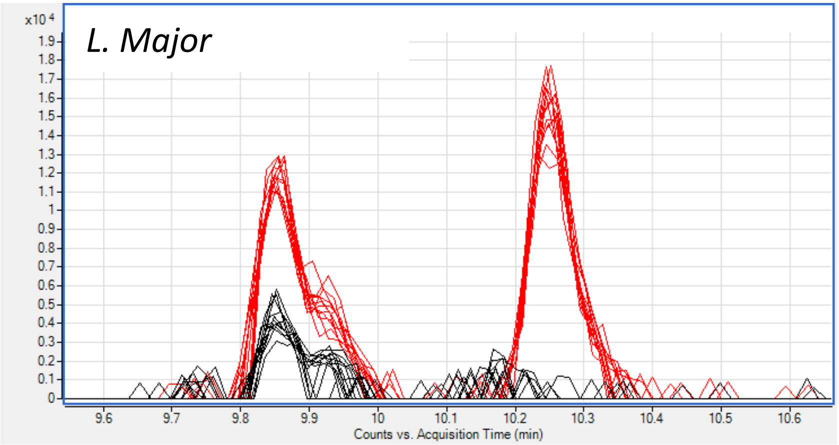

Experimental *m/z* 369.3524 corresponding to water loss of 386.3549, mass of one sterol in pathway

Single peak identified in each species corresponding to cholesterol

Cholesterol

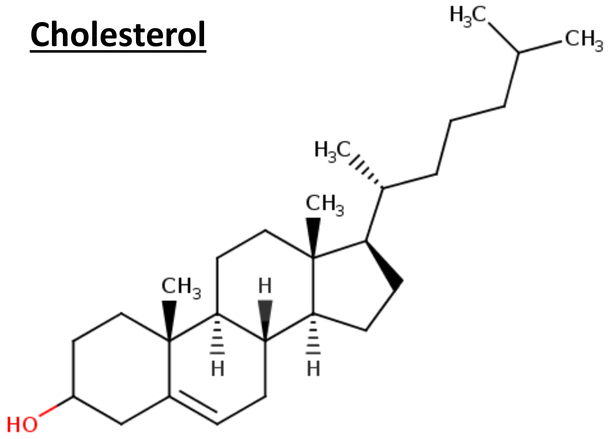

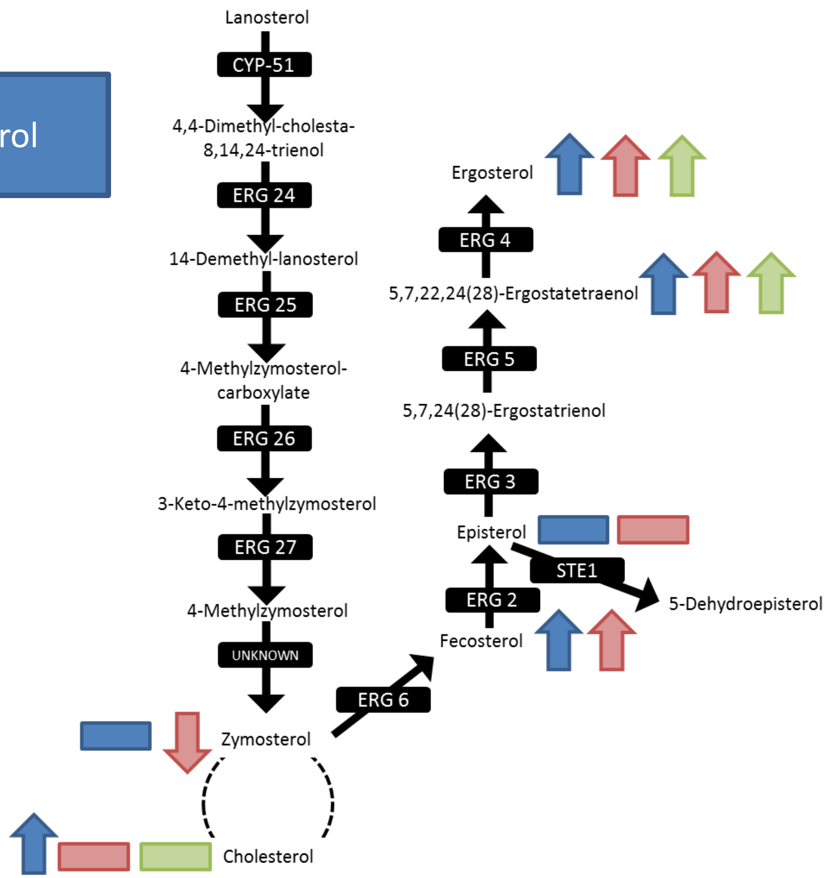

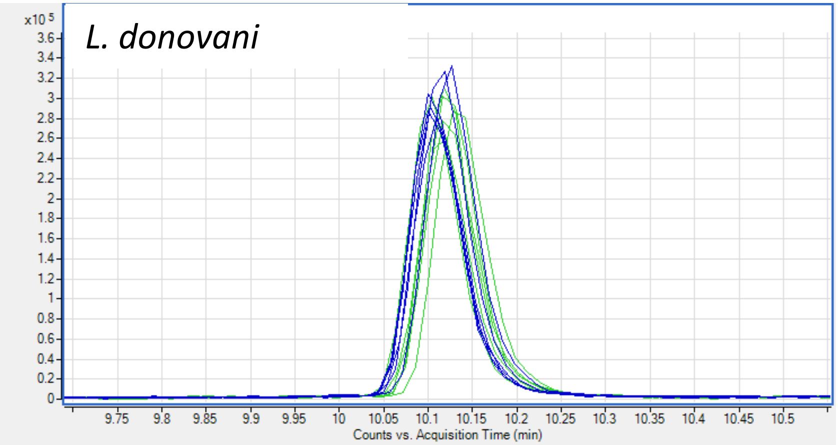

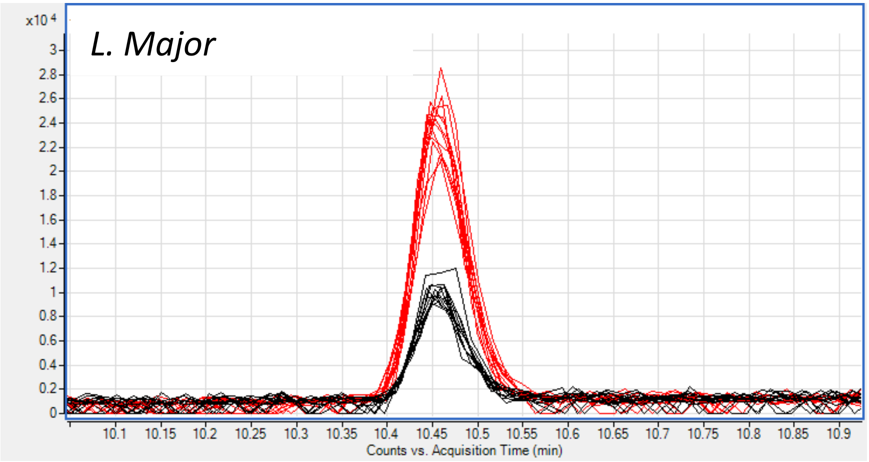

Experimental *m/z* 367.3364 corresponding to water loss of 384.3392, mass of one sterol in pathway

Single peak identified in *L.donovani* corresponding to zymosterol.
Peak not present in *L.major* data

Zymosterol

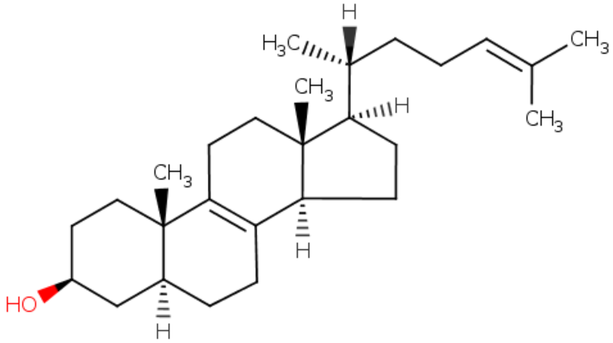

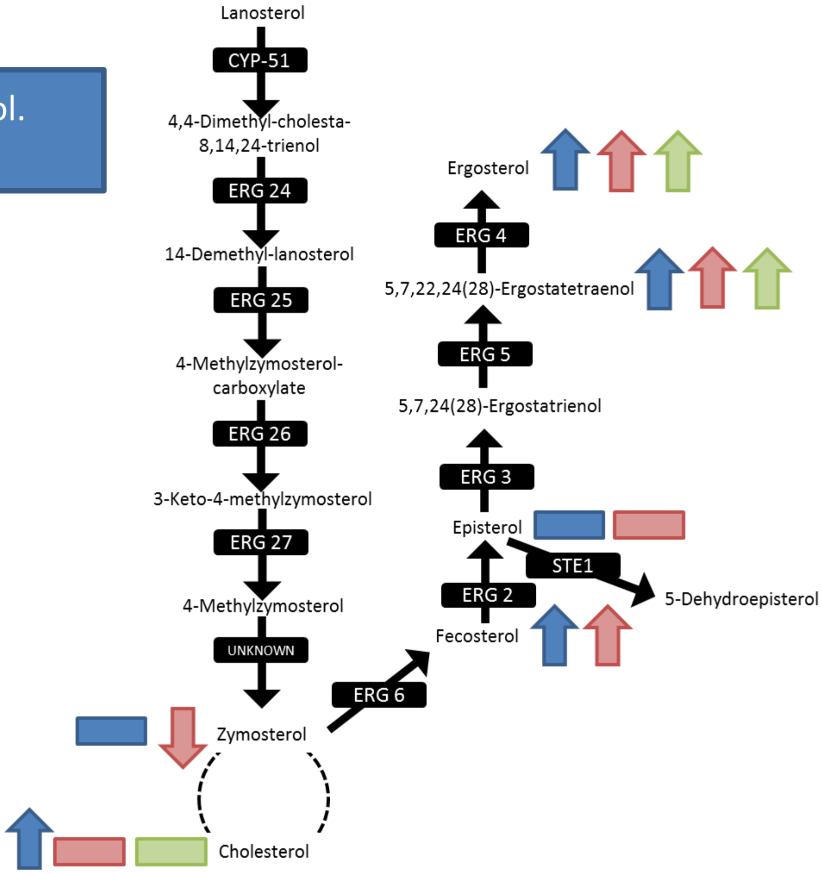

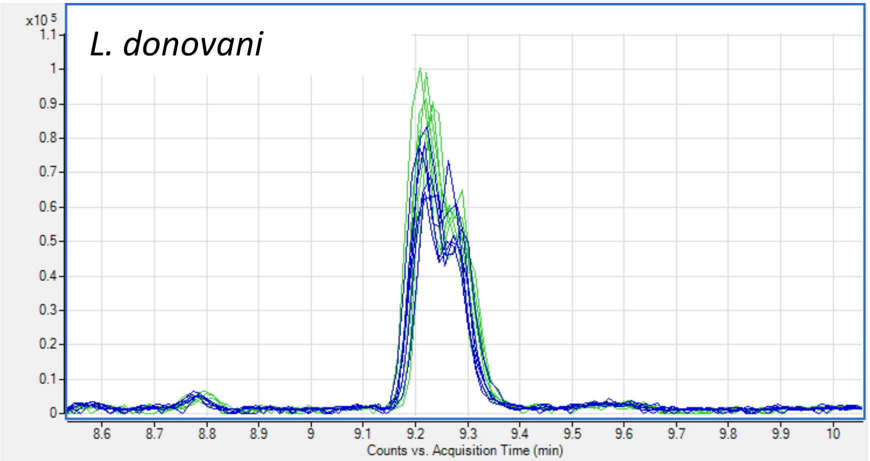

L. Major

Not detected

Experimental *m/z* 379.3352 corresponding to water loss of 396.3392, mass of four sterols in pathway (highlighted)

3-keto4-methylzymosterol was discounted since it has no possibility for neutral loss of water in structure. Three peaks identified in both species. First determined to be ergosterol by LogP calculation. Second or third could correspond to 5,7,24(28)-Ergostatrienol / 5-Dehydroepisterol, unable to say which due to them having the same LogP.

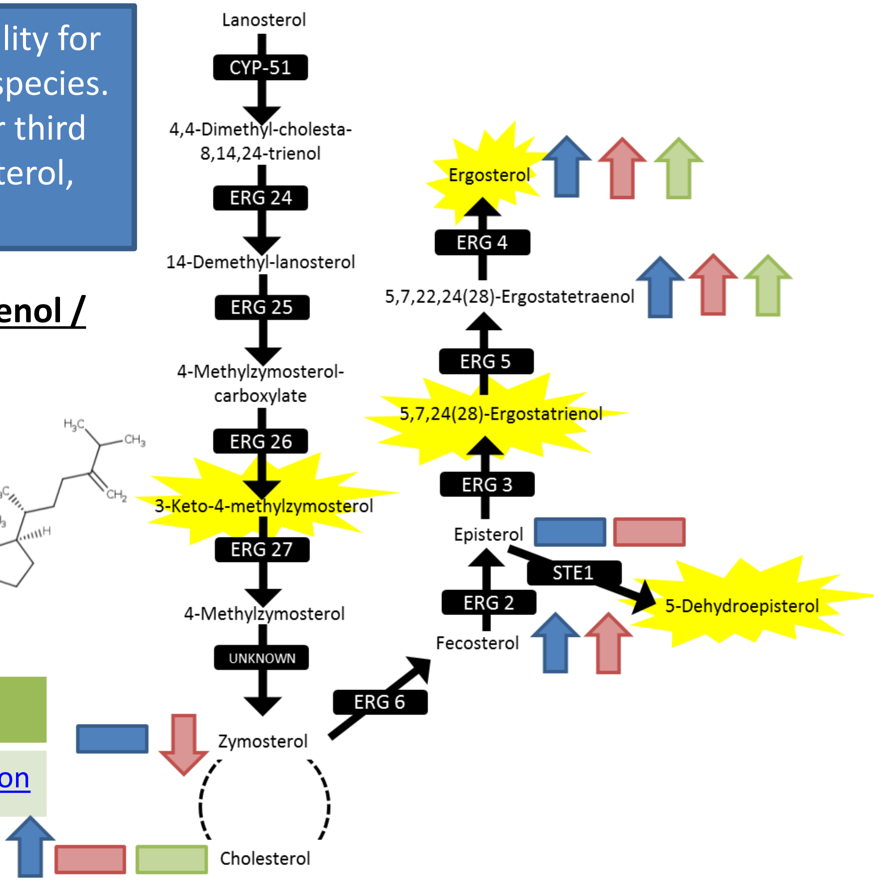

3-keto-4-methylzymosterol

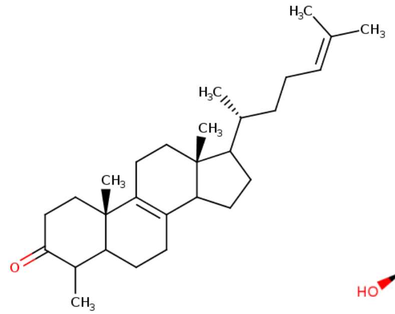

Ergosterol

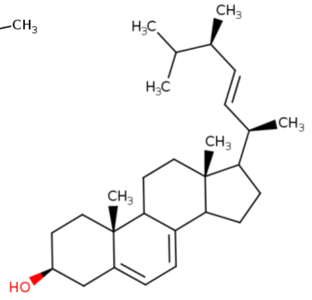

logP	7.39	ALOGPS
logP	6.63	ChemAxon

5,7,24(28)-Ergostatrienol / 5-Dehydroepisterol

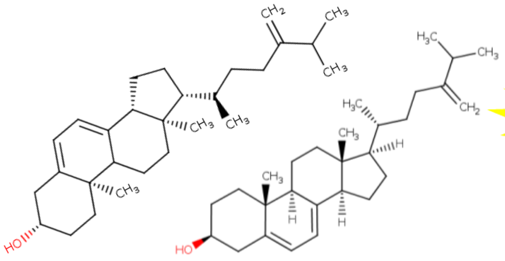

logP	7.64	ALOGPS
logP	6.65	ChemAxon

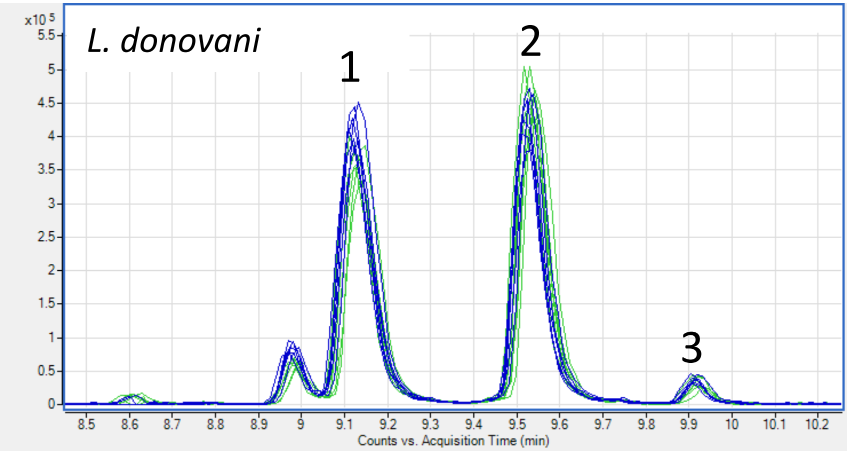

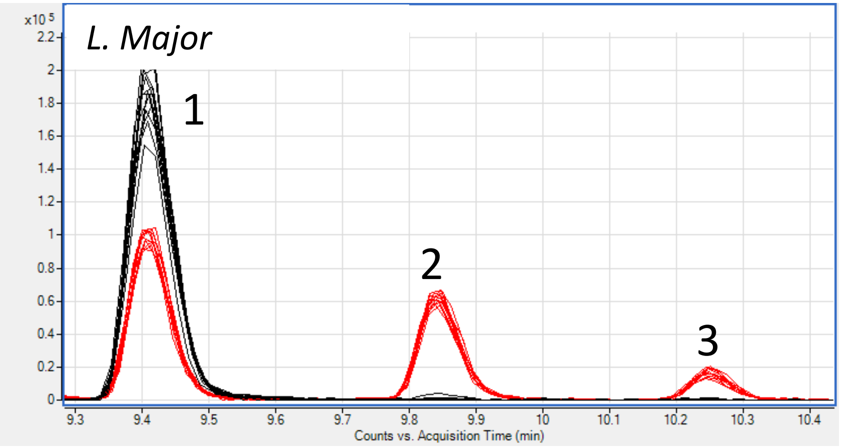

Experimental *m/z* 377.3202 corresponding to water loss of 394.3236, mass of one sterol in pathway

Although there are a number of peaks in the raw data, there is only one possibility in the pathway. After filtration and statistical analysis only the second peak as identified remained in datasets and this was putatively assigned to be 5,7,22,24(28)-Ergostatetraenol although this identification could belong to one of the other peaks that was rejected.

5,7,22,24(28)-Ergostatetraenol

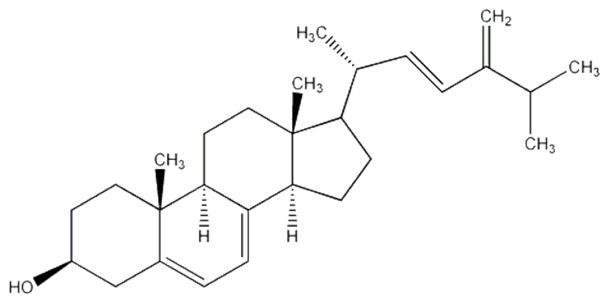

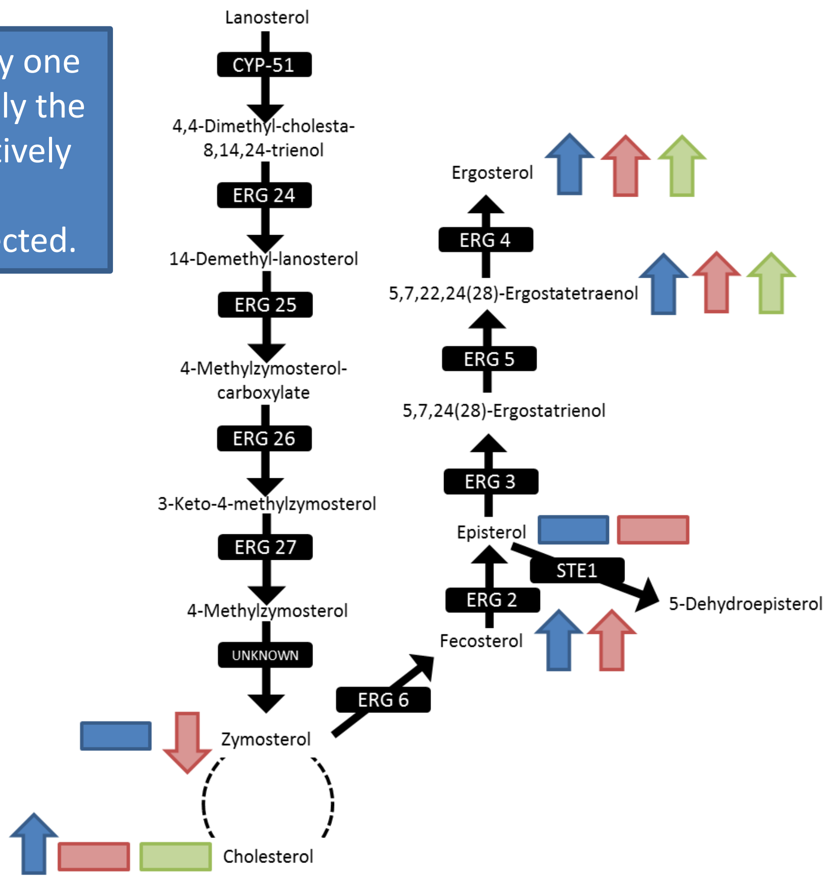

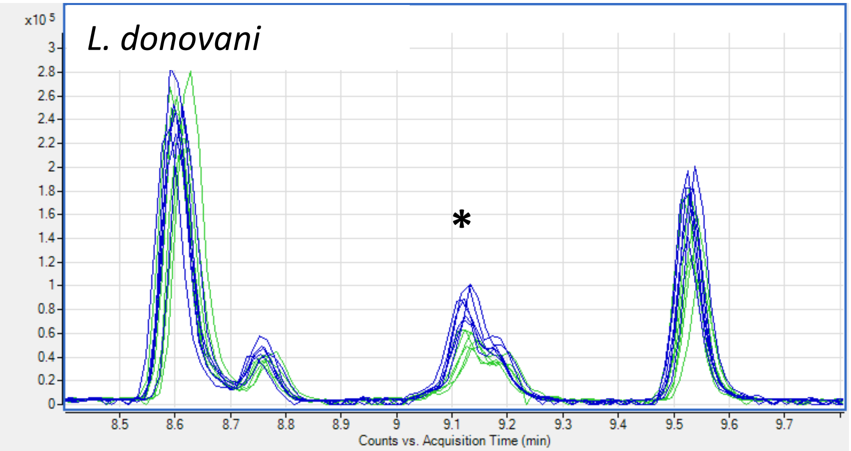

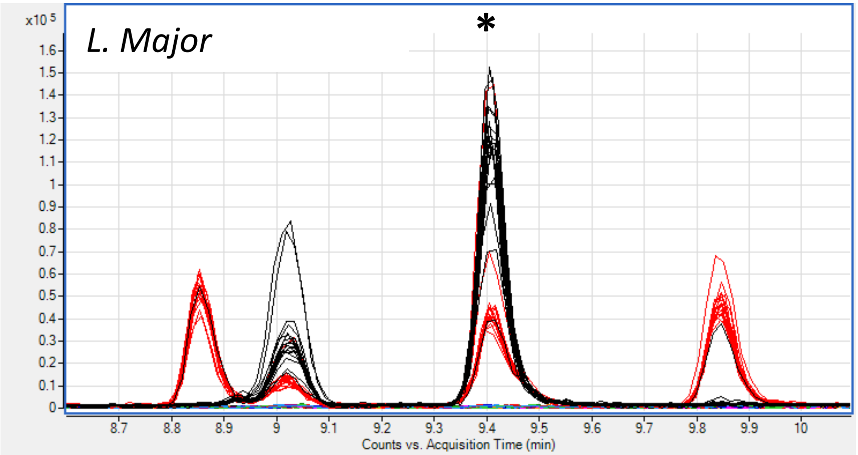

Supplement: Supplemental material [file AAC.02095-17_zac005187095s1.pdf]
